# The Effects of Primary Tumor Location on Survival after Liver Resection for Colorectal Liver Metastasis in the Mediterranean Population

**DOI:** 10.3390/jcm12165242

**Published:** 2023-08-11

**Authors:** Ahmad Mahamid, Omar Abu-Zaydeh, Esther Kazlow, Dvir Froylich, Muneer Sawaied, Natalia Goldberg, Yael Berger, Wissam Khoury, Eran Sadot, Riad Haddad

**Affiliations:** 1 Department of Surgery, Carmel Medical Center, Haifa 3436212, Israel; mahamidam@yahoo.com (A.M.); oabuzaydeh@gmail.com (O.A.-Z.); ekazlow@gmail.com (E.K.); dvirfr7@gmail.com (D.F.); moneerswaed@gmail.com (M.S.); wekhoury@gmail.com (W.K.); 2 The Ruth and Bruce Rappaport Faculty of Medicine, Technion-Israel Institute of Technology, Haifa 3200003, Israel; natalia.goldberg@gmail.com; 3 Department of Radiology, Carmel Medical Center, Haifa 3436212, Israel; 4 Department of Surgery, Rabin Medical Center, Petch Tikvah 4941492, Israel; yaelberger1@gmail.com (Y.B.); eransadot@gmail.com (E.S.); 5 Sackler School of Medicine, Tel Aviv University, Tel Aviv 6139001, Israel

**Keywords:** colorectal liver metastasis, survival, colon cancer, tumor side

## Abstract

(1) Background: There is an abundance of literature available on predictors of survival for patients with colorectal liver metastases (CRLM) but minimal information available on the relationship between the primary tumor location and CRLM survival. The studies that focus on the primary tumor location and CRLM survival exhibit a great deal of controversy and inconsistency with regard to their results (some studies show statistically significant connections between the primary tumor location and prognosis versus other studies that find no significant relationship between these two factors). Furthermore, the majority of these studies have been conducted in the West and have studied more diverse and heterogenous populations, which may be a contributing factor to the conflicting results. (2) Methods: We included patients who underwent liver resection for CRLM between December 2004 and January 2019 at two university-affiliated medical centers in Israel: Carmel Medical Center (Haifa) and Rabin Medical Center (Petach Tikvah). Primary tumors located from the cecum up to and including the splenic flexure were labeled as right-sided primary tumors, whereas tumors located from the splenic flexure down to the anal verge were labeled as left-sided primary tumors. (3) Results: We identified a total of 501 patients. Of these patients, 225 had right-sided primary tumors and 276 had left-sided primary tumors. Patients with right-sided tumors were significantly older at the time of liver surgery compared to those with left-sided tumors (66.1 + 12.7 vs. 62 + 13.1, *p* = 0.002). Patients with left-sided tumors had slightly better overall survival rates than those with right-sided tumors. However, the differences were not statistically significant (57 vs. 50 months, *p* = 0.37 after liver surgery). (4) Conclusions: The primary tumor location does not significantly affect patient survival after liver resection for colorectal liver metastasis in the Mediterranean population.

## 1. Introduction

Colorectal cancer with liver metastasis (CRLM) is a major health concern worldwide. CRLM occurs in almost half of all patients with colorectal cancer, with an estimated 20–25% of newly diagnosed colorectal cancer patients presenting with liver metastases at the time of diagnosis [1]. 

According to the Israeli Health Ministry, colorectal cancer has become the second most prevalent malignancy in Jewish and Arab Israeli women, closely following breast cancer. It is the second most common malignancy in Arab Israeli men following lung cancer, and the third most prevalent malignancy in Jewish Israeli men following prostate and lung cancers. According to an international comparison, the incidence rate of colorectal cancer is slightly higher in Israel in comparison to the world average [2]. There are various factors that influence the prognosis of patients with CRLM. A largely understudied factor is the relationship between the primary tumor location (right colon versus left colon) and prognosis in patients with CRLM. The literature available on this association has provided conflicting and inconsistent results.

A previous study identified seven factors as significant and independent predictors of poor long-term outcomes. These included a positive margin, extrahepatic disease, node-positive primary disease, a disease-free interval from primary to metastasis < 12 months, a number of hepatic tumors > 1, the largest hepatic tumor > 5 cm, and a carcinoembryonic antigen level > 200 ng/mL. A preoperative scoring system was developed using the last five criteria, assigning one point for each criterion. The total score was highly predictive of outcomes (*p* < 0.0001). No patient with a score of 5 or above was a long-term survivor [3]. Other studies have identified reliable prognostic biomarkers in patients with resectable CRLM. In a 2013 study, Umeda et al. analyzed genomic DNA obtained from CRLM tissue from patients undergoing curative hepatic resection. The results showed that KRAS and BRAF mutations were poor prognostic factors in CRLM and that microsatellite instability (MSIH) cancer rarely revealed metastatic potential [4].

A 2015 retrospective study investigated the prognostic impact of the primary tumor location in metastatic colorectal cancer (mCRC) in patients receiving first-line chemotherapy ± bevacizumab in three independent cohorts: a prospective pharmacogenetic study (PROVETTA) and two randomized phase III trials, AVF2107g and NO16966. The study found that patients with left-sided primary tumors had superior OS (*p* < 0.001) and progression-free survival (*p* < 0.001) in comparison to patients with right-sided primary tumors [5].

Recent studies also found that patients with CRLM with right-sided primary tumors had a significantly worse prognosis compared to those with left-sided primary tumors [6,7]. Moreover, other studies have suggested that right-sided colon cancers are more likely to have specific genetic mutations that may contribute to their more aggressive behavior. One such mutation is the BRAF mutation, which has been found to be more common in right-sided colon cancers compared to left-sided colon cancers [8].

However, other studies have reported conflicting results. For example, a 2011 study analyzed the relationship between the colon cancer location (right versus left side) and tumor stage and their effects on 5-year mortality. The study found that there was no major difference in mortality between right- and left-sided cancers for all stages. Stage II right-sided cancers had lower mortality than left-sided cancers, while stage III right-sided cancers had higher mortality than left-sided tumors [9]. Furthermore, a 2017 study investigated the impact of the primary tumor location on overall survival (OS), recurrence-free survival (RFS), and long-term outcomes in patients undergoing potentially curative resection of colorectal liver metastases (CRLM). The analysis was based on data from a single-institution database of 907 patients who underwent initial resections for CRLM. The results showed that patients with left-sided primary tumors had a significantly improved median OS compared to those with right-sided primaries (5.2 years vs. 3.6 years, *p* = 0.004). However, there was no significant difference in median RFS stratified by primary location (1.3 vs. 1.7 years, *p* = 0.065) and the association of the primary location with RFS was not statistically significant in a multivariable analysis (*p* = 0.105) [10].

It is important to note that most of the current data on the differences between right- and left-sided colon cancers come from studies conducted in the Western world. There are limited data available on the differences between these tumors in the Mediterranean population, and it is unclear whether the same differences exist in this population. The aim of our study was to investigate, for the first time, this largely understudied association with a geographically specific population with the aim of improving the interpretability and validity of this association so that it can be further extrapolated to the population at large. By studying this homogenous population, we hoped to gain a better understanding of the genetic and environmental factors that contribute to the differences in prognosis between right- and left-sided colon cancers. This may ultimately lead to more personalized and effective treatments for patients with CRLM.

## 2. Materials and Methods

Patients who underwent liver resection for CRLM between December 2004 and January 2019 were identified from the surgical databases at Carmel Medical Center (Haifa, Israel) and Rabin Medical Center (Petach Tikvah, Israel). Clinical indicators of these patients’ perioperative courses were retrospectively examined, including their demographics, detailed surgical history, pathology results, and oncologic follow-up records. The study was conducted in accordance with the Declaration of Helsinki and Good Clinical Practice Guidelines and was approved by the institutional review boards (IRBs) of the Carmel and Rabin Medical Centers. Indications for surgery were determined during a weekly multidisciplinary conference. The pre-operative workup included blood tests, tumor markers, imaging modalities (computed tomography (CT), positron emission CT (PET-CT), and magnetic resonance imaging (MRI)), and characterization of the specific tumor (number, location, size, and relation to intrahepatic vascular or biliary structures). All patients underwent standard evaluation for surgery by an attending anesthesiologist. Patients were informed in detail about the procedure, including the risks and benefits, and written consent was obtained before surgery. 

Metastasis was defined as the occurrence of a liver tumor, during follow-up or at diagnosis, in patients with colorectal cancer. Tumors located from the cecum up to and including the splenic flexure were labeled as right-sided primary tumors. Tumors located from the splenic flexure down to the anal verge were labeled as left-sided primary tumors. Based on previous studies, rectal cancer patients were treated as left-sided primary tumors in our study [5]. Blood loss was estimated using the volume of blood lost from the abdominal cavity during the procedure. Operative time was defined as the time elapsed from the initial incision until closure. Postoperative hospital stay was defined as the number of hospitalized days from the day of operation until the day of discharge, inclusive. We used the Clavien–Dindo grading system to characterize any post-operative complications occurring within 30 days of surgery [11]. Tumor size and resection margins were determined according to the pathological reports from the permanent sections of tissue samples. R0 was defined as no cancer cells seen microscopically at the resection margin.

After discharge, the patients were followed by our multidisciplinary team during the first month post-surgery, every 4 months for the first 2 years thereafter, and subsequently twice a year. Follow-up examinations included blood work (including CBC, chemistries, liver function tests, and carcinoembryonic antigens) and a spiral CT of the chest–abdomen or PET-CT as indicated.

### Statistical Analysis 

All statistical analyses were performed using IBM statistics (SPSS) vs. 24. Continuous variables were summarized with the mean ± SD or median and IQR, as appropriate. Categorical variables were presented as numbers and proportions. Disease-free (DFS) and overall survival (OS) were estimated using Kaplan–Meier curves and compared between groups by log-rank test. *p* < 0.05 was considered statistically significant.

## 3. Results

**Figure 1:**
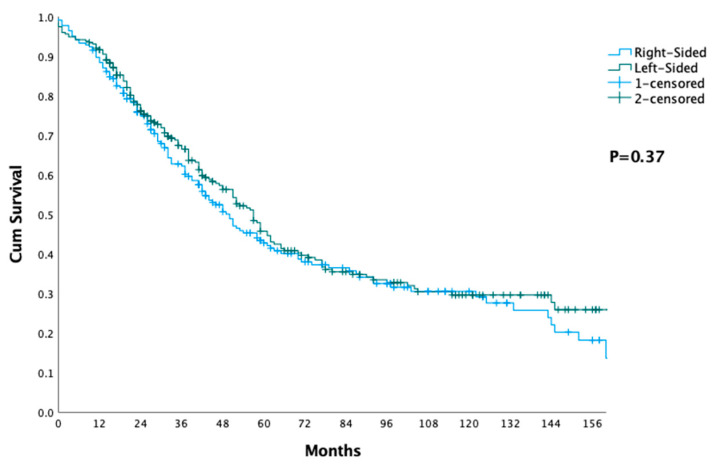
Overall survival after liver surgery.

**Figure 2:**
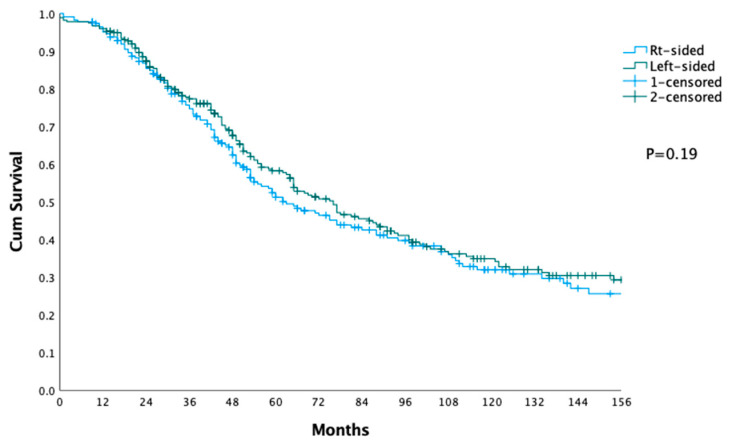
Overall survival after colon surgery.

The data from Table 1, Table 2 and Table 3 and Figure 1 and Figure 2 provide a detailed analysis of the differences and similarities between patients who underwent liver surgery for CRLM, as categorized by the primary tumor location.

Table 1 shows that patients with right-sided primary tumors were significantly older (mean age 66.1 ± 12.7 years) than those with left-sided primary tumors (mean age 62 ± 13.1 years) (*p* = 0.002). The gender distribution was similar between the two groups (*p* = 0.43). In terms of T stage, there were no significant differences between the groups (*p* = 0.19). The distribution of *n* stages was also similar between the two groups, with no major differences observed (*p* = 0.20). The percentage of patients who had disease-free survival (DFS) for liver metastases of more than 12 months was not appreciably different between the two groups (*p* = 0.35).

Clinical risk factors (Fong), the size of the largest liver tumor, and the number of liver metastases were not significantly different between the two groups (*p* = 0.31, *p* = 0.08, *p* = 0.24, respectively). There was no noteworthy difference in R status between the two groups (*p* = 0.16). The proportion of patients who received perioperative chemotherapy was not significantly different between the two groups (*p* = 0.27). However, more patients in the right-sided group received blood transfusions (*p* = 0.02) and experienced complications after surgery (*p* = 0.053). The distribution of liver resection types (anatomical and non-anatomical) and the sequence of resection were also not significantly different between the two groups (*p* = 0.37 and *p* = 0.31, respectively).

Table 2 and Figure 1 show the short- and long-term outcomes after liver surgery, comparing the survival between the groups at different time points (12, 24, 36, 60, 120 months). The median survival time for the right-sided group was 50 months (95% CI 41:60), and for the left-sided group, it was 57 months (95% CI 47:58). There were no major differences in survival rates between the two groups (*p* = 0.37).

Table 3 and Figure 2 show the short- and long-term outcomes after colon surgery. Comparing the survival between the groups at different time points (12, 24, 36, 60, 120 months), the median survival time for the right-sided group was 63 months (95% CI 49:77), and for the left-sided group, it was 76 months (95% CI 63:89). There were no significant differences in survival rates between the two groups (*p* = 0.19).

## 4. Discussion

Our study suggests that the primary tumor location does not significantly affect patient survival after liver resection for colorectal liver metastasis in the Mediterranean population.

There has been a great deal of controversy with regard to the primary tumor location and the ultimate prognosis of patients with CRLM available in the literature to date. The majority of these studies have been conducted in the West and focused on heterogenous populations, which may be a contributing factor to the conflicting results. The aim of our study was to investigate, for the first time, this relationship (primary tumor location and prognosis) in a Mediterranean population. Our goal was to study a more homogenous population in an attempt to minimize the potential for false-positive or false-negative results related to genetic confounding. 

A 2017 systematic review and meta-analysis aimed to determine the prognostic role of the primary tumor location in patients with colon cancer. The review included 66 studies with a total of 1,437,846 patients and concluded that a left-sided primary tumor location was associated with a significantly reduced risk of death independently of stage, race, adjuvant chemotherapy, year of study, number of participants, and quality of included studies. The study suggested that the colon cancer location should be acknowledged as a criterion in establishing prognosis at all stages of the disease and should be considered when deciding on a treatment in metastatic settings [12].

Furthermore, a 2020 review discussed the impact of the primary tumor location on the prognosis of patients after the local treatment of CRLM. The authors reviewed and analyzed 10 studies that examined the association between right- and left-sided colorectal cancer on overall survival (OS) and recurrence-free survival (RFS) after local treatment (resection and/or ablation) of CRLM. The results showed that patients with right-sided tumors had a significantly decreased OS (*p* < 0.001) and RFS (*p* = 0.03) compared to patients with left-sided tumors [6]. 

Another study conducted in 2019 investigated the impact of the location of the primary tumor on the long-term survival outcomes of colorectal liver metastases following hepatic resection. The review analyzed 12 studies with 6387 patients and found that primary right-sided colorectal liver metastases following hepatic resection had significantly worse overall survival compared to left-sided tumors. However, the analysis did not find a significant difference in disease-free survival [7].

The role of tumor genetics in relation to the primary tumor location and prognosis is a potential confounding variable. A study on data from twins in Sweden, Denmark, and Finland analyzed the role of genetic and environmental factors in the development of cancer. The study found that genetic factors play a minor role in the development of most types of cancer and that environmental factors have the primary role in causing sporadic cancer. However, the study also revealed that genetics play a significant role in the development and prognosis of prostate and colorectal cancer [13].

Tsilimigras et al. conducted a systematic review of the literature in 2018, which analyzed the clinical significance and prognostic relevance of genetic mutations of KRAS for resectable and unresectable CRLM. The review included 78 studies that reported mutation data for patients with resectable and unresectable CRLM. The review found that KRAS mutation was a negative prognostic factor for overall and recurrence-free survival [14] Studies have also shown that the BRAF mutation is associated with worse prognosis and an increased risk of recurrence in patients with CRLM [8,14,15]. Furthermore, Yaeger et al. found that BRAF-mutant CRLM cases were associated with a right-sided location [8]. Interestingly, the BRAF mutation has been found to be more common in Western populations [16]. Therefore, the lower prevalence of BRAF mutations in the Mediterranean population may contribute to the lack of significant differences in survival between right- and left-sided primary tumors in our study.

The reason for these conflicting results Is not entirely clear, but one possible explanation is that the studies used different definitions of the primary tumor location, leading to inconsistent results. Another possible explanation is that the studies included heterogenous patient populations with varying characteristics, such as age, gender, and disease stage, which may have contributed to the differences in the results. In addition, genetic differences between populations may also contribute to the conflicting results on the impact of the primary tumor location and the prognosis of patients with CRLM. The Mediterranean population differs from Western populations in many genetic and environmental factors, such as a high degree of endogamy, climate, and diet, which may play a role in the differences in tumor biology and clinical outcomes.

The impact of the primary tumor location on the prognosis of patients with colorectal liver metastasis (CRLM) remains controversial. While several studies have investigated the relationship between the primary tumor location and patient outcomes, the results have been inconsistent. In this study, we investigated the effects of the primary tumor location on the survival of patients who underwent liver resection for CRLM in the Mediterranean population. Our findings showed no statistically significant differences between patients who had right-sided and left-sided primary tumors in terms of overall survival after liver resection for CRLM. Our results are consistent with previous studies that have reported no significant differences in long-term survival and recurrence-free survival between right- and left-sided primary tumors in patients with CRLM.

Our study has several limitations that should be considered when interpreting the results. Firstly, the retrospective nature of the study may have introduced selection bias, as only patients who underwent liver resection for CRLM were included in the analysis. Furthermore, we did not analyze the genetic differences between right and left colon cancers in our study, which may have contributed to our findings. Additionally, our study focused on the Mediterranean population, and the results may not be applicable to other populations. Lastly, another potential confounding variable is age. In our study, patients with right-sided tumors were significantly older at the time of liver surgery compared to those with left-sided tumors. Despite these limitations, our study has several strengths, including the analysis of a homogeneous population of patients from the Mediterranean region, which reduces the confounding effects of genetic and environmental factors that may vary across different populations. In addition, our study provides evidence of the impact of the primary tumor location on the prognosis of patients with CRLM, which is a topic of ongoing debate in the literature. Lastly, our study adds to the growing body of literature on the differences between right and left colon cancers, and the implications for the clinical outcomes of patients with CRLM.

## 5. Conclusions

Overall, our study suggests that the primary tumor location may not be a significant predictor of survival in patients who undergo liver resection for CRLM in the Mediterranean population. However, further research is needed to better understand the genetic and environmental factors that contribute to the differences in prognosis between right- and left-sided colon cancers across different populations, and to develop personalized treatment strategies for patients with CRLM based on their tumor characteristics.

## Figures and Tables

**Table 1:** Baseline demographic, liver, and colorectal characteristics, and perioperative and histological outcomes.

	Right-Sided Primary (Total = 225) *n* (%)	Left-Sided Primary (Total = 276) *n* (%)	*p*
Age (liver surgery)	66.1 ± 12.7	62 ± 13.1	0.002
Gender			0.43
Male	129 (57.3%)	155 (56.2%)	
Female	96 (42.7%)	121 (43.8%)	
T			0.19
1	1 (0.4%)	3 (1.1%)	
2	7 (3.1%)	14 (5.1%)	
3	157 (69.8%)	144 (52.2%)	
4	8 (3.6%)	13 (4.7%)	
unknown	52 (23.1%)	102 (36.9%)	
N			0.20
N0	61(27.1%)	75 (27.2%)	
N1	72 (32%)	64 (23.2%)	
N2	43 (19.1%)	34 (12.4%)	
unknown	49 (21.8)	103 (37.3%)	
DFS (liver mets)			0.35
<12 months	126 (56%)	142 (51.4%)	
>12 months	89 (39.6%)	110 (38.9%)	
unknown	10 (4.4%)	24 (8.7%)	
Clinical risk factor			0.31
0–2	156 (69.4%)	154 (55.8%)	
3–5	48 (21.3%)	41 (14.9%)	
unknown	22 (9.3%)	81 (29.3%)	
Size of largest liver tumor			0.08
<5 cm	201 (89.3%)	223 (80.8%)	
>5 cm	24 (10.7%)	53 (19.2%)	
Number of liver mets			0.24
1	117 (52%)	152 (55.1%)	
>1	108 (48%0	124 (44.9%)	
R status			0.16
Negative	207 (92%)	245 (88.7%)	
Positive	18 (8%)	31 (11.3%)	
Peri-operative chemotherapy			0.27
Yes	155 (68.9%)	197 (71.4%)	
No	70 (31.1%)	79 (28.6%)	
Blood transfusion			0.02
Yes	41 (18.2)	67 (24.3%)	
No	176 (78.2%)	178 (64.5%)	
unknown	8 (3.6%)	31 (11.2%)	
Liver resection			0.37
Anatomical	43 (19.1%)	67 (24.4%)	
Non-anatomical	182 (80.1%)	208 (75.6%)	
Complication			0.053
Yes	100 (44.4%)	100 (36.2%)	
No	125 (55.6%)	176 (63.8%)	
Sequence of resection			0.31
Liver first	9 (3.6%)	19 (6.6%)	
Colon first	181(80.7%)	213 (77.2%	
Combined	35 (15.7%)	44 (16.2%)	

**Table 2:** Short- and long-term outcomes after liver surgery.

	Right-Sided Primary (Total = 225)	Left-Sided Primary (Total = 276)	*p*
12 months	88.4%	91.6%	
24 months	74.9%	76.2%	
36 months	62.3%	66.5%	
60 months	42.8%	45.8%	
120 months	30.6%	29.6%	
Median (95% CI) months	50 (41:60)	57 (47:58)	0.37

**Table 3:** Short- and long-term outcomes after colon surgery.

	Right-Sided Primary (Total = 225)	Left-Sided Primary (Total = 276)	*p*
12 months	96.4%	96.7%	
24 months	86.8%	87.3%	
36 months	74.7%	77.3%	
60 months	51.3%	57.8%	
120 months	32%	34.2%	
Median (95% CI) months	63 (49:77)	76 (63:89)	0.19

## Data Availability

The data presented in this study are available on request from the corresponding author.
